# Electronic and optoelectronic applications of solution-processed two-dimensional materials

**DOI:** 10.1080/14686996.2019.1669220

**Published:** 2019-09-19

**Authors:** Jingyun Wang, Bilu Liu

**Affiliations:** Shenzhen Geim Graphene Center (SGC), Tsinghua-Berkeley Shenzhen Institute (TBSI), Tsinghua University, Shenzhen, P. R. China

**Keywords:** Solution processed 2D materials, assembly, sensors, electronics, optoelectronics

## Abstract

The isolation of graphene in 2004 has initiated much interest in two-dimensional (2D) materials. With decades of development, solution processing of 2D materials has becoming very promising due to its large-scale production capability, and it is therefore necessary to examine progress on solution-processed 2D materials and their applications. In this review, we highlight recent advances in the assembly of solution-processed 2D materials into thin films and the use of them for electronics and optoelectronics. We first present an overview about typical approaches to assemble solution-processed 2D materials into desired structures, including layer-by-layer assembly, Langmuir–Blodgett assembly, spin coating, electrophoretic deposition, inkjet printing, and vacuum filtration. Then, electronic and optoelectronic applications of such assembly films are presented, such as thin-film transistors, transparent conductive films, mechanical and chemical sensors, photodetectors and optoelectronic devices, as well as flexible and printed electronics. Finally, our perspectives on challenges and future opportunities in this important field are proposed.

## Graphical Abstract

**Figure:**
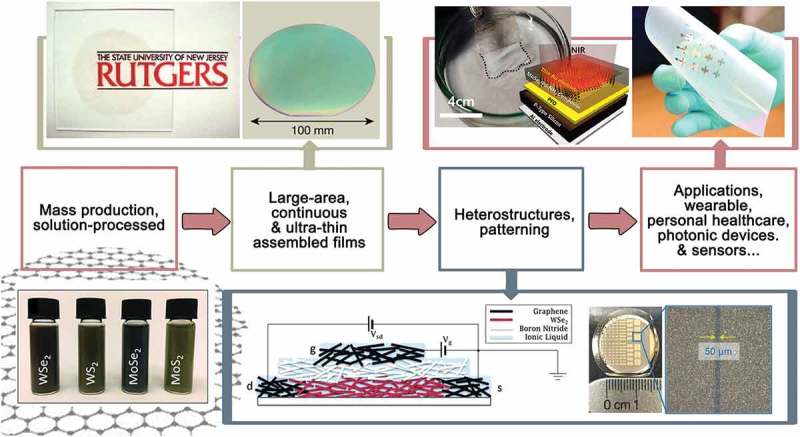

## 1. Introduction

Two-dimensional (2D) materials have become a significant class of materials since Geim, Novoselov, and co-workers isolated graphene from graphite by the scotch tape exfoliation method in 2004 [1]. Due to its outstanding properties such as ultra-high carrier mobility [1,2], high optical transparency and thermal conductivity [3,4], high Young’s modulus [5], graphene has drawn substantial research interest and shown applications in different areas [6–9]. The unexpected properties of graphene have inspired researchers to explore other 2D materials with similar layered structures but versatile properties, which greatly enriches the 2D materials family[2]. Besides graphene, other 2D materials like hexagonal boron nitride (h-BN), graphitic carbon nitride (g-C_3_N_4_), transition metal dichalcogenides (TMDCs), metal oxides, black phosphorus (BP), and MXenes have also been extensively studied these years. Importantly, these different kinds of 2D materials can be metallic (e.g., graphene, MXenes, some TMDCs), semiconducting (e.g., some other TMDCs, metal oxides, BP) or insulating (e.g., h-BN, silicates), and possess very different optical properties [10–15]. Such diverse electronic and optoelectronic properties of 2D materials, in together with their unique 2D planar structures, make them suitable building blocks for various electronics, optoelectronics, and many other applications [7,16–19].

Materials preparation is the first prerequisite for their applications. For the case of 2D materials, bottom-up synthetic and top-down exfoliation are two strategies to prepare them. In the content of bottom-up synthesis, chemical vapor deposition (CVD) is one of the most widely used methods by which high-quality 2D materials can be controllably grown [20,21]. In addition, atomic layer deposition (ALD) and molecular beam epitaxy (MBE) [22] have also been utilized to grow 2D materials, which may lower the growth temperature and reduce the vapor pressure of chalcogen precursors during TMDC growth. Although the bottom-up synthetic methods especially CVD growth have been widely used to prepare 2D materials, significant efforts have also been devoted on the development of top-down exfoliation methods to prepare 2D materials. The basic work principle of these top-down methods is to exfoliate layered materials from their bulk counterparts under the assistance of certain forces such as sonication, ball milling, shearing force, intercalation-induced weakening of interlayer interaction, etc. [21]. These top-down exfoliation methods have the following advantages. First, they have potential to produce 2D materials in large scale and low cost, which is critical for the fast-growing demands of printed and flexible electronics and other applications [23]. Second, top-down exfoliated 2D materials are solution-processible, and can be assembled into large-area and designed structures under low temperatures on arbitrary substrates, providing important foundations for applications. Third, exfoliation could be a universal method to prepare many 2D materials, while some of them are not easy to be made by bottom-up synthetic methods currently, such as mica, layered double hydroxides, BP. Therefore, top-down exfoliation strategy can produce various 2D materials with large quantity, solution-processibility, and show promising for practical applications. Besides, some 2D materials and oxides layered materials can also be synthesized by hydrothermal method and sol-gel method [24–26], which are two other methods to get solution-processed 2D materials.

In this review, we start from solution-processed 2D materials, and discuss their assembly engineering and the state-of-the-art electronics-related applications. Since from solution-processed 2D materials to 2D materials-based electronics and optoelectronics, the assembly plays the key role, we first summarize various assembly techniques to prepare thin films from 2D building blocks. The main evaluation criteria of these assembly methods are discussed. Then, based on these assembly techniques, we introduce some typical electronic-related applications of assembled 2D structures, such as thin-film transistors (TFTs), transparent conductive films (TCFs), mechanical and molecule sensors, photodetectors, and optoelectronics, as well as flexible and printed electronics. Finally, current challenges in this field are discussed, and our thoughts on the solutions to these challenges and future opportunities are presented.

## 2. Assembly of solution-processed 2D materials into thin films

**Figure 1.**
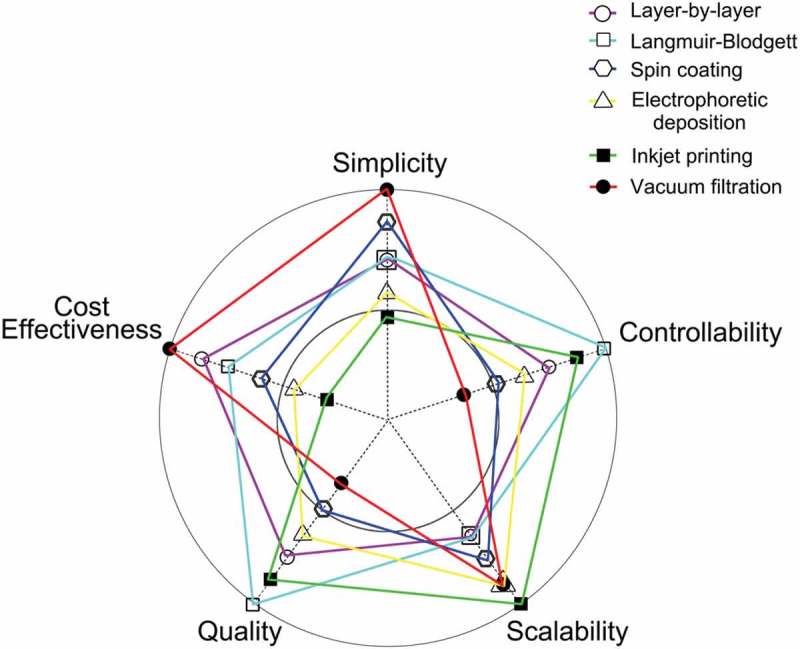
Comparisons of six assembly methods to fabricate thin films of 2D materials. The comparison criteria include cost-effectiveness, simplicity, controllability, scalability, and quality of assembled film.

Highly integrated electronics or optoelectronics with solution-processed 2D materials usually consist of different functional films. However, controllable growth of these films is still a big challenge by conventional growth approaches. Film manufacturing as well as film assembly of solution-processed 2D materials have become an important technique. Therefore, for electronic and optoelectronic applications of 2D materials, it is important to have the pre-designed thin-film structures assembled by 2D material building blocks. Many efforts have been devoted on the assembly of 2D materials for further electronic-related applications [27,28], like 2D material array electronics, which can cater well to low-cost, high-density-integration and highly sensitive sensing systems. Therefore, we will briefly describe and compare six typical methods including layer-by-layer assembly, Langmuir–Blodgett technique, spin coating, electrophoretic deposition, inkjet printing, and vacuum filtration for assembly of 2D materials into thin-film structures, and propose five important criteria to evaluate these assembly methods, including cost-effectiveness, simplicity, controllability, scalability, and quality of assembled film (Figure 1).

Layer-by-layer assembly method is based on the electrostatic deposition of charged 2D materials. It was invented for the alternative adsorption of positively and negatively charged polyelectrolytes, which can achieve the deposition of nanoscale conductors, semiconductors, and dielectrics [29,30]. Simplicity and the variety of components are two key advantages of this method. The concentration of dispersion used in layer-by-layer assembly process needs to be less than 0.1g/L to ensure a good stability of charged 2D materials [21]. Since Kovtyukhova et al. produced multilayer films by alternative adsorption of anionic graphite oxide sheets and cationic poly(allylamine hydrochloride) (PAH) in the 1990s [31], layer-by-layer method has been regarded as one of the most commonly used approaches to assemble 2D materials and various other low-dimensional materials.

As for the Langmuir–Blodgett assembly method, the history can be traced back to the 1930s when Irving Langmuir and Katherine Blodgett fabricated a monolayer of amphiphilic molecules on water surface [32,33]. It was first invented for the preparation of monomolecular films of fatty acid [34]. The basic work principle of the Langmuir–Blodgett method is that amphiphilic molecules can form a monolayer on water surface, and during slowly dip-coating process, the monolayer can be transferred onto a solid substrate. Now, it has expanded its application in the assembly of ultra-thin film (monolayer or multilayer) of different kinds of components. It has also been widely used in the self-assembly of nanoparticles or other complex components to construct a variety of organized ultra-thin films [35,36]. The Langmuir–Blodgett method has good controllability, and is simple and scalable. Meanwhile, it can achieve precise control of the layer thickness, yield scalable and homogeneous deposition of layers, and allows a dense packing of monolayer of soft (organic) or hard (inorganic) materials with good density and alignment. Currently, 2D materials are also used as building blocks to fabricate ultra-thin films with high-density through the Langmuir–Blodgett method. For example, Li et al. prepared a film of graphene by Langmuir–Blodgett assembly, which is highly transparent and conductive [37]. In another work, Cote et al. have systematically analyzed the Langmuir–Blodgett assembled graphene oxide (GO) films with two fundamental geometries, i.e., edge-to-edge and face-to-face, and pointed out that the GO monolayer can be used as a great candidate for electronic applications [36]. It is clear that the Langmuir–Blodgett assembly is a promising method to prepare thin films of solution-processed 2D materials with high-quality.

Compared with layer-by-layer and Langmuir–Blodgett methods, spin coating is a relatively convenient approach for large-scale fabrication of thin films. In this method, 2D materials suspension will be firstly dropped onto a target substrate. Then, a certain rotation speed and time will be used to spread the suspension and prepare a film with equal thickness around all substrate surfaces. Spin-coating method has several advantages like simplicity, fast, and high efficiency. Many 2D materials have been assembled by this method into thin films. For example, Vendamme et al. prepared ultra-thin films of titania (TiO_2_) and other metal oxides by spin-coating process [38]. Recently, Matsuba et al. developed a one-pot spin-coating method and fabricated dense monolayer thin films made of various 2D materials within 1 min over a very wide area (30-mm-diameter) [39]. The ultra-thin films prepared by spin-coating can be used to fabricate TFT devices. However, if one wants to prepare thick films, it is better to choose other assembly approaches rather than spin-coating, because it is difficult to ensure the uniformity of thick films in spin-coating method which would involve complicated cycling process and dispersion formula.

Electrophoretic deposition (EPD) is a colloidal movement process of charged materials in suspension under an extra electric field. This feature indicates that the deposition process will occur if the suspended materials are electrically charged. Fortunately, solution-processed 2D materials are usually positively or negatively charged, which makes EPD work. During the EPD process, 2D materials or other colloids need to be well dispersed in the suspension to ensure the stability of the colloidal system. Different from the methods mentioned above, which are usually used to fabricate thin films, EPD can easily achieve large-scale assembly of thick films on substrates. The equipment to perform EPD is low-cost and simple. In addition, the deposition rate is controllable, and the deposition kinetics is predictable. 2D materials with lateral sizes less than 50 µm can be easily driven by electrical field and deposited by EPD. GO is one of the first 2D materials which has been assembled by EPD [40]. Various deposition morphologies such as free-standing films, wrinkle films, and multi-layer 2D composites can be obtained by modulating current change during EPD process. There are many studies utilize EPD method for the fabrication of heterostructures or patterned structures of 2D materials. For example, Lin et al. have fabricated a MoS_2_-graphene composite film by EPD, which has high transmittance (>70%) under visible light [41]. It is noted that chemical reactions such as reduction or aqueous electrolysis may happen during EPD process, which would bring some side-effects toward the assembled structure.

Inkjet printing is another powerful method to fabricate thin films of 2D materials for printed electronic and energy devices [10,42]. The basic work principle is that suitable inks of 2D materials can be directly written on substrates under control of computer. This technique can fabricate different pre-patterned structures. For solution-processible 2D materials, inject printing is a promising way to fabricate complicated, multi-functional and scalable electronic devices and circuits. One major concern of inkjet printing is the ink optimization because many inks and solvents currently used are toxic and costly. In order to make the whole printing process more controllable and stable, lots of efforts have been made on optimizing the properties of the solvents and the types of surfactants. Ideal solvent should have several traits, including good dispersion of high concentration 2D materials, low-cost, and low-toxicity. Recently, Secor et al. have developed an environmentally friendly graphene ink containing ethanol and ethyl cellulose to fabricate large-area, conductive and flexible electronics [43]. One of the most straightforward GO inks based on water was developed in 2011 [42,44]. Researchers demonstrated that monolayer GO with average size of 500 nm × 500 nm can be dispersed in water to make a stable ink for inkjet printing. In addition, McManus et al. reported a water-based solvent to make inks of many 2D materials including graphene, MoS_2_, WS_2_ and h-BN [45]. The advantages of inkjet printing technology include ability of direct printing 2D materials with designed patterns, good spatial resolution of about 5 µm, and good universality of the method to print most 2D materials. Thus, inkjet printing will become an important technique for future 2D materials-based flexible electronic applications. However, the disorder of printed 2D materials in thin films may be a limitation in their applications for certain purposes where alignment is the requirement.

Vacuum filtration is another commonly used method to fabricate thin films of 2D materials [46,47]. First, 2D materials dispersion is prepared. Then, vacuum pump is used to suck the dispersion to go through a filter. After solvent passing through the filter, the film or membrane composed of 2D materials will form. Among all the assembly methods mentioned in this review, vacuum filtration may be the fastest and the easiest one to prepare large-area films. Therefore, the advantages of vacuum filtration include simple and low-cost setup, as well as fast process. In addition, it can be used to easily prepare relatively thick 2D film, and the filtrate can be peeled off from the filter to become a large and freestanding film. However, if the film is several hundred of micrometers thick, blocking effect will occur which will make the vacuum filtration process very slow, and if the film is thinner than tens of nanometers, a procedure to transfer it to desired substrate is needed. This feature somehow limits the application of vacuum filtration on high-quality film fabrication.

As shown in Figure 1 and Table 1, each method has its own advantages and shortcomings, and the choice of assembly method should be related to the specific applications targeted.

**Table 1.** A summary of different kinds of assembly methods for 2D film construction.

Assembly method	Advantages	Disadvantages
Layer-by-layer	Controllable thickness Simple Suitability for large-area assembly	Time consuming for thick films Polyelectrolyte in dispersion will influence film quality 2D materials must be charged
Langmuir-Blodgett	Simple High controllability during film assembly process High-quality of ultra-thin film Non-selective for most 2D materials	Time-consuming Assembly size cannot be very large
Spin coating	Simple Fast Controllable thickness	Limited thickness of thin film Difficult to ensure a uniform film on a large area 2D materials should be charged for high-quality films
Electrophoretic deposition	Fast Can achieve large-area thick film	2D materials must be charged Chemical reactions will make some side-effect during the whole process Hard to precisely control the thickness
Inkjet printing	Desired patterned printing for flexible device applications can be achieved. Good spatial resolution Suitable for most 2D materials	Printing instruments are relatively complicated and expensive Very high demand for 2D materials inks
Vacuum filtration	Simple and fast Can prepare free-standing films Suitable for most 2D materials	Substrate is limited Extra transfer process is needed Hard to prepare thick films

## 3. Electronic and optoelectronic applications of assembled 2D material thin films

Assembled structures of solution-processed 2D materials especially the thin-film form provide great potential in electronics and optoelectronics. As for electronic properties, 2D materials could be insulating (e.g., h-BN), semiconducting (e.g., TMDCs, BP), or metallic (e.g., graphene, MXenes). As for optical properties, bandgaps of 2D materials cover a wide electromagnetic spectra range from far infrared to deep UV, which is attractive for optoelectronics. In this section, we will discuss recent developments of electronic and optoelectronic applications using solution-processed 2D materials including TFTs, TCFs, mechanical and molecule sensors, photodetectors and optoelectronics, flexible and printed electronics. First, we present two basic applications, TFTs and TCFs. Then, different sensors based on solution-processed 2D materials are discussed. Later, photodetectors and optoelectronics by solution-processed 2D materials as well as their basic principles are discussed. Finally, we review recent achievements in using 2D materials for flexible and printed electronics.

### 3.1. TFTs

The first TFT can be dated back to 1960s, when Weimer demonstrated the first polycrystalline cadmium sulfide (CdS) thin-film based field-effect transistors[48]. TFTs are composed of multilayers including three electrodes (source, drain, and gate) as well as a dielectric layer, and its principles is resembling metal–semiconductor field-effect transistor, in which the current between source and drain is modulated by regulating the carrier injection in the interface between channel and dielectric layer through the gate voltage. However, compared with integrated circuits which usually use FETs with short channel lengths (~10-nanometer node now), channel lengths in TFTs are usually large, e.g., tens or hundreds of micrometers. Also, the mobility of TFT is normally around 0.1–100 cm^2^V^−1^s^−1,^ which is lower than that in high-performance FETs in integrated circuits[49]. Therefore, TFTs especially amorphous and polycrystalline silicon-based TFTs are largely used in display applications. Though a-Si TFTs have already matured, there still exist some clear research motivations. The first one is to reduce the cost, because big display is expensive but a big market. The second motivation is flexible displays or devices. Thus, it is highly desirable to develop and investigate new material systems, which could make big breakthrough towards TFTs. Solution-processed 2D material is such a promising candidate that a lot of research has begun to focus on this area.

**Figure 2.**
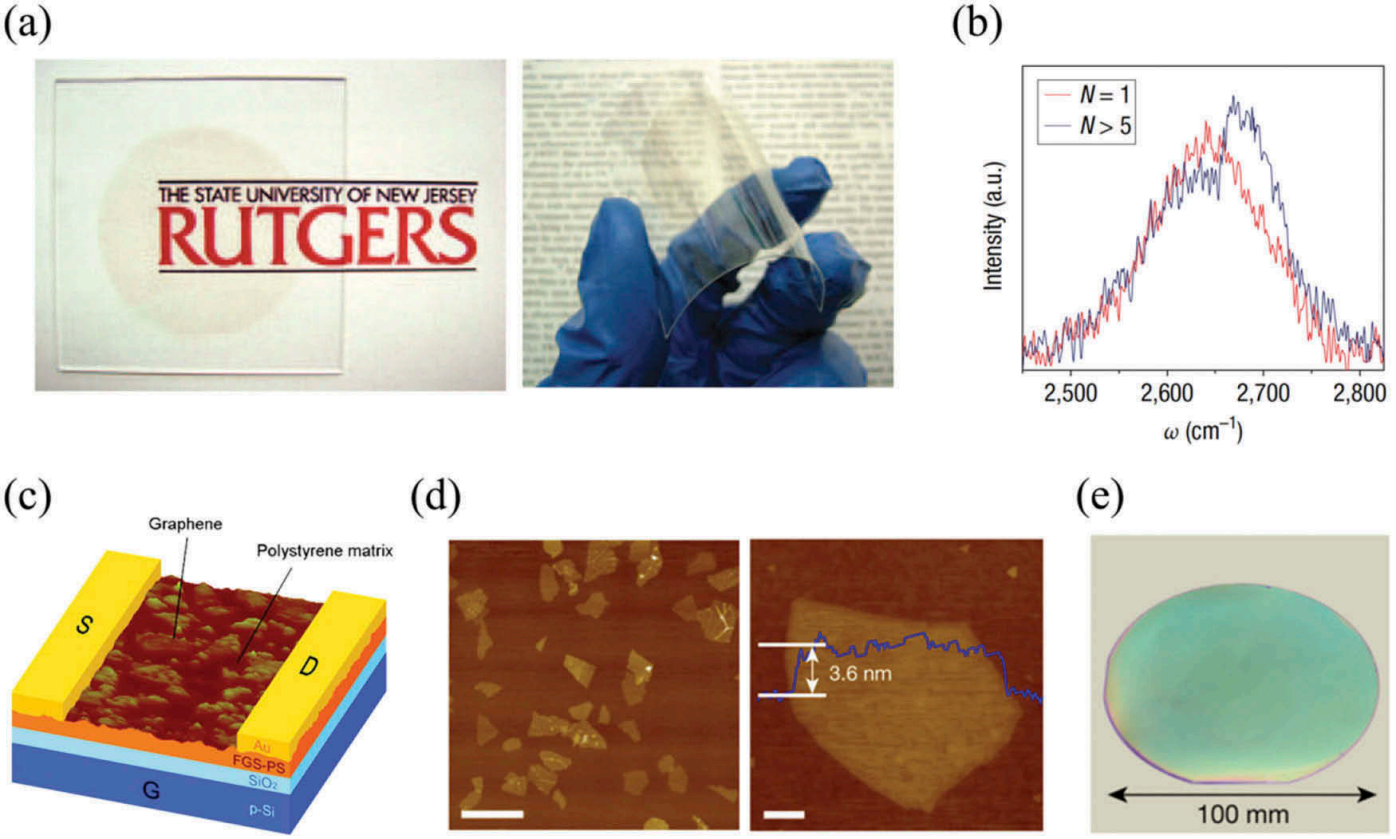
Illustration of different solution-processed 2D materials-based TFTs and their properties. (a) Photographs of transparent GO thin films on glass and photographs of flexible GO thin films on plastic substrates. (b) The 2D peak in Raman spectra for a single-layer GO thin film (N = 1) and for a multiple-layer GO thin film (N > 5). (c) Schematic of FGS-PS composite TFTs. (d) Left: AFM image of MoS_2_ nanosheets. Scale bar, 2 μm. Right: AFM image of an individual MoS_2_ nanosheet. (blue line: height profile across the nanosheet). Scale bar, 200 nm. (e) Photograph of the MoS_2_ thin film on a SiO_2_/Si wafer. (a-b) Reprinted from ref 50. Copyright © 2008, Springer Nature (c) Reprinted from ref 52. Copyright © 2009, American Chemical Society. (d-e) Reprinted from ref 56. Copyright © 2018, Springer Nature.

Graphene and its derivatives like GO or reduced GO (rGO) are the pioneer 2D materials being studied to fabricate TFTs. Eda et al. reported a vacuum-filtration assembly method for the deposition of rGO ultra-thin films [50]. As shown in Figure 2(a), the films are transparent and flexible over a large area of 10 cm^2^. Besides, the thicknesses of such films can range from monolayer to over five layers (Figure 2(b)). The electrical and optical properties of the films can be modulated over six orders of magnitude. The fabricated TFTs showed graphene-like ambipolar behavior with hole and electron mobilities of 1 and 0.2 cm^2^ V^−1^ s^−1^, respectively. In 2012, Lee et al. have developed an all graphene-based TFTs deposited on flexible plastic substrates by Langmuir–Blodgett method [51]. The graphene and GO layers acted as the active layer and dielectric layer together with the graphene electrodes. The hole and electron mobilities of this all graphene-based TFT were 300 and 250 cm^2^ V^−1^ s^−1^, respectively. Not only the pure graphene or rGO but also graphene-based composite thin films can be utilized in TFTs. For example, Eda et al. have reported a solution-processed polystyrene (PS) functionalized graphene sheets (FGS) based TFT with ambipolar characteristics and semiconducting behavior (Figure 2(c)) [52]. These results indicated that the percolating or functionalization of graphene network with some insulating polymers will render graphene to be semiconducting. Subsequently, with the exploration of other kinds of 2D materials, TFTs based on TMDCs, metal oxides or other 2D materials have greatly advanced the development of electronic devices. For example, TFT made of MoO_3_ nanoflakes has shown a hole mobility of 600 cm^2^ V^−1^ s^−1^ and an I_on_/I_off_ ratio of ~3 × 10^5^ [53]. This I_on_/I_off_ ratio is much higher than that in the graphene-based TFTs thus enlarging its applications in digital electronics. TMDCs are another type of 2D materials applied in TFTs. Xi et al. reported solution-processed method to fabricate MoS_2_ thin films [54]. The substrate was first introduced into a homogeneous solution. Then, the solution temperature was increased to 90°C for 27 min to deposit MoS_2_ thin film. By this simple method, the MoS_2_ thin film can achieve selective deposition with a thickness of 11 nm. The carrier mobility of TFTs made by this method is 0.4 cm^2^ V^−1^ s^−1^. In another work, MoS_2_ from a two-step liquid-phase exfoliation (LPE) method was sprayed onto the substrates as channels in TFTs [55]. The carrier mobility of this TFT was 10^−4^ cm^2^ V^−1^ s^−1^, a relatively low value. It seems that TFTs made by solution-processed 2D materials usually possess poor performance as shown in Table 2. This is because that the exfoliated 2D materials have more defects with small flake size and broad thickness distribution, which leads to poor quality of materials and unsatisfactory performance of electronics. However, these problems can be overcome by developing high-quality synthesis solution-based method and suitable solution assembly method which can not only achieve ultra-thin, high-quality and uniform 2D films but also possess low-cost and scalable fabrication capability. Based on this, Duan’s group reported an electrochemical intercalation method to prepare high-quality 2D materials, for example, MoS_2_ (Figure 2(d)) with a mobility of 10 cm^2^V^−1^s^−1^ which is much higher than that of Li–exfoliated MoS_2_ nanosheets [56]. Besides, the mobility of TFTs constructed by this MoS_2_ large-area and thin film were improved to about 10 cm^2^ V^−1^s^−1^ as shown in Figure 2(e).

**Table 2.** The mobility summary of different TFTs systems by solution-processed 2D materials.

Materials systems of TFTs	Mobility (cm^2^V^−1^s^−1^)	Ref.
Graphene based thin-film	Holes: 1 Electrons: 0.2	50, 51
	Holes: 300 Electrons: 250	
MoO_3_ thin-film	600	53
MoS_2_ thin-film	0.4	54, 55, 56
	10^−4^	
	10

In contrast to a large number of publications on the use of CVD grown 2D materials for TFTs [57], research on solution-processed 2D materials TFTs is still at its early stage. Optimizing the solution-based exfoliation methods and exploring new assembly methods are two important goals in this field [58]. In short, solution-processed 2D material-based TFTs are of great research value for large-scale and reliable production of some low-resolution displays. For example, a TFT from solution-processed 2D materials with a mobility of 1 cm^2^V^−1^s^−1^ can be used to drive liquid crystal displays (LCDs), which do not need rather high carrier mobilities like OLEDs (>10 cm^2^ V^−1^s^−1^) [49].

### 3.2. TCFs

TCFs refer to transparent conductive films, which means that the materials should have high optical transparency usually in visible light wavelength and good electrical conductivity. Therefore, 2D materials from solution-processed method can easily satisfy the first requirement as they have a quite low absorption in visible light range when they are exfoliated to be less than a few nanometers. For example, monolayer graphene with one atom thick only has a 2.3% absorption of incident white light[3]. For the second requirement, good electrical conductivity, graphene, and its derivatives are representative 2D materials because of their high conductivity.

**Figure 3.**
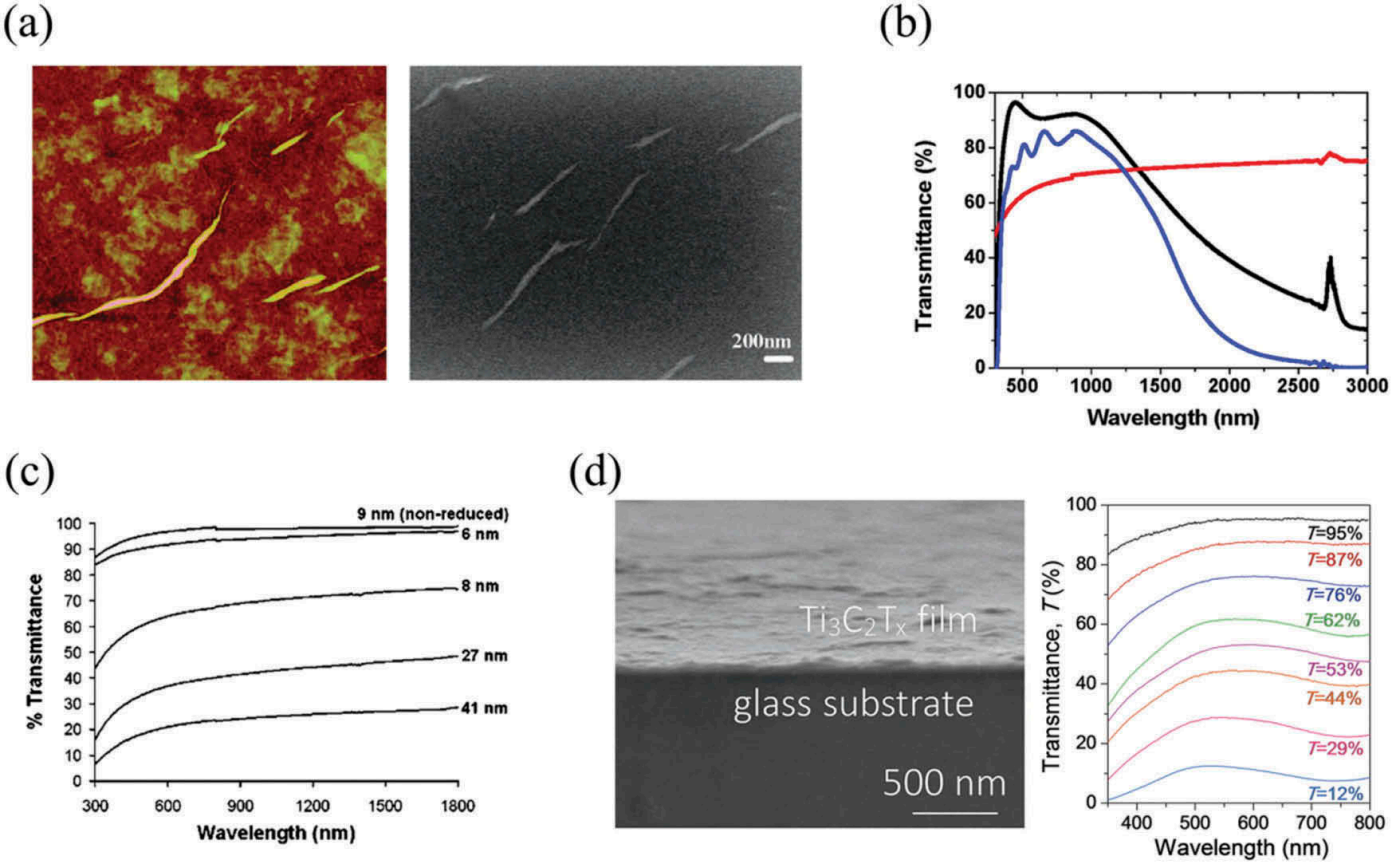
Illustration of different solution-processed 2D materials based TCFs and their performance. (a) AFM image (3.2 × 3.2 µm^2^) (color scale: black to bright yellow, 30 nm) and SEM image of GO film. (b) Transmittance of a 10 nm thick graphene film (red), in comparison with that of indium tin oxide (black) and fluorine tin oxide (blue) glasses. (c) Optical transmittance spectra of rGO films with the film thickness increasing. (d) SEM images of spun-cast Ti_3_C_2_T_x_ film, and transmittance spectra of Ti_3_C_2_T_x_ films with different thicknesses. (a-b) Reprinted from ref 60. Copyright © 2008, American Chemical Society. (c) Reprinted from ref 61. Copyright © 2008 American Chemical Society. (d) Reprinted from ref 62. Copyright © 2017 WILEY-VCH Verlag GmbH & Co. KGaA, Weinheim.

Hence, the earliest trial of 2D materials-based TCFs is naturally made by graphene. Watcharotone et al. reported the pioneer graphene-based TCFs in 2007 [59]. A solution-processed method was explored to directly coat the graphene-based conductive film onto the pre-selected transparent substrate, silica. They employed water-soluble GO as a filler so that the GO could completely fill the vacancy within organic polymers. Because GO could be rendered conductive by chemical reduction, the composite matrix would become conductive. With the highest loading (11 wt%) of GO, the TCF showed a high transmittance of 95% at 550 nm. However, the corresponding conductivity was relatively low about 0.45 ± 0.06 S/cm. Almost at the same time, GO sheets made by Hummers method were also deposited onto the substrates and were reduced into graphene to make transparent and conductive films by Wang et al. (Figure 3(a)) [60]. The film exhibited a conductivity of 500 S/cm. As shown in Figure 3(b), a 10 nm thick graphene film has a transparency of more than 70% at 550 nm compared with that of indium tin oxide and fluorine tin oxide glasses. In another work, Becerril et al. reported an all-GO transparent conductive film [61]. They used spin-coating to deposit GO thin-films on quartz. Compared with chemical reduction methods, they draw a conclusion that a high-temperature graphitization treatment is more effective for removing functionalized groups in GO and improving its conductivity. Such high temperature treated rGO films had a conductivity of ~10^2^ S/cm together with a transparency of greater than 80% under 400–1800 nm, as shown in Figure 3(c). The properties of high electrical conductivity and low optical absorption allowed graphene to be an excellent candidate for next-generation transparent and conductive film applications. Besides graphene, Titanium carbide MXene (Ti_3_C_2_T_x_) has also been reported as transparent conductive 2D systems. Zhang et al. demonstrated a highly transparent and conductive Ti_3_C_2_T_x_ film (Figure 3(d)) which was fabricated by spin-casting of Ti_3_C_2_T_x_ nanosheet colloidal solutions. The Ti_3_C_2_T_x_ film (4 nm) showed transmittance of 93% at 550 nm and conductivity of 5736 S/cm, and with the film thickness increasing, the transmittance of Ti_3_C_2_T_x_ based TCF decreased as shown in Figure 3(d) [62].

Some other materials such as silver nanowires, carbon nanotubes and poly(3,4-ethylenedioxythiophene (PEDOT) also have good conductivity and high transparency in visible range, which are used in TCF as well [63–67]. In comparison, the transmittance data of different material systems were summarized in Table 3. Most of the 2D material TCF systems can reach a transmittance of 80% with a conductivity of 10^2^ S/cm. It is also worth mentioning that some large-area TCFs by CVD growth graphene have already been commercialized [68]. Bae et al. reported a roll-to-roll CVD production process and prepared 30-in. graphene films with sheet resistances of about 125 Ω sq^−1^ and monolayer optical transmittance of 97.4% at 550nm [69]. It means that 2D materials especially graphene have the potential to compete or complement convention TCF materials systems, for example, indium tin oxide (ITO). However, the large-area films produced from CVD method usually show inferior performance compared with convention ITO films but a higher cost. How to realize high-quality and large-area uniform 2D materials films and how to build these films on arbitrary substrates are two challenges in this field. Therefore, the high-quality solution-processed 2D materials together with subtle assembly methods may be a suitable solution to this problem especially in future flexible and wearable devices area.

**Table 3.** A summary of different TCFs by solution-processed 2D materials.

TCFs systems	Transmittance (%) under 550nm	Conductivity (S/cm)	Ref.
rGO	95	0.45	59, 60, 61
	70	500	
	80	100	
2D Ti_3_C_2_T_x_	93	5736	62
RuO_2_/PEDOT:PSS	93	279	63
silver nanowire	50	6.472	65
carbon nanotube	60	733	67

### 3.3. Mechanical and molecule sensors

**Figure 4.**
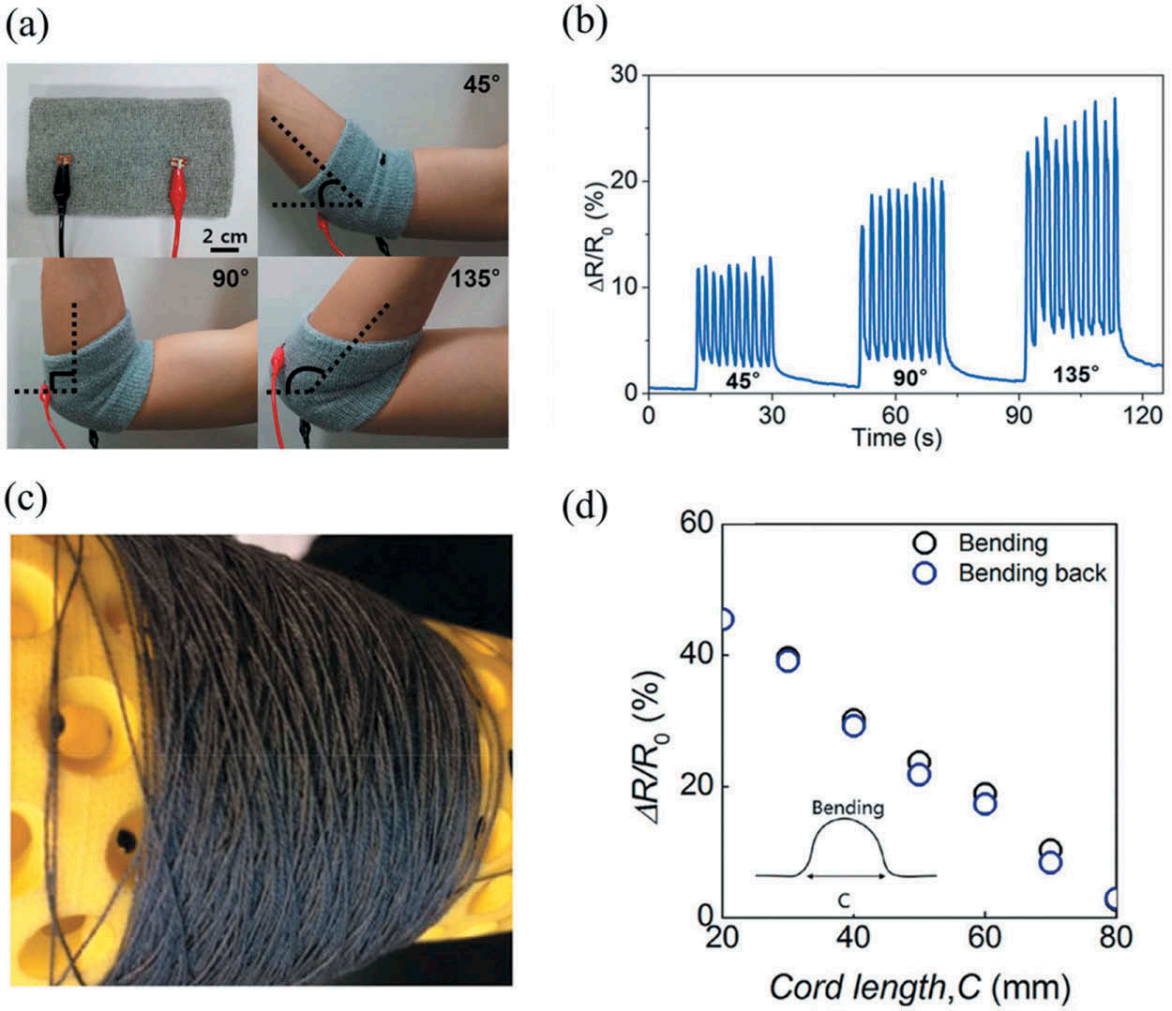
Pressure or strain sensors using solution-processed 2D materials as active materials. (a) Photographs showing elbow wrap based on the yarn strain sensors to monitor the arm bending motion at different angles of 45°, 90°, and 135°. (b) Relative resistance change of the yarn strain sensor according to the bending motions. (c) Hank of rGO dyed cotton yarns. (d) Resistance change of graphene yarn sensors when bending in forward (concave downward) and in reverse (concave upward) directions. (a-b) Reprinted from ref 70. Copyright © 2015 American Chemical Society. (c-d) Reprinted from ref 71. Copyright © 2019 American Chemical Society.

Due to their unique structures, multifunctionalities, and easy assembly feature, solution-processed 2D materials can be applied for different kinds of electrical-based sensors, including pressure or strain sensors, gas sensors, and biosensors. Among them, pressure or strain sensors are one of the most typical sensors. Response time, signal linearity, and sensitivity are three main features of merits to evaluate the performance of sensors. In general, a sensor with short response time, broad-range linearity and high sensitivity is ideal. Graphene and its derivatives are the most commonly used 2D materials for pressure or strain sensors. Compared with CVD graphene-based pressure sensors, the sensors from solution-processed graphene can achieve the scale-up production, low-cost fabrication, and easy assembly. Based on that, Park et al. employed a layer-by-layer assembly method to realize a stretchable and flexible graphene-based strain sensor [70]. In this work, yarn was used as template. The dispersion of chemically exfoliated graphene nanoplatelets was coated onto the yarns by layer-by-layer assembly. The strain sensor with intertwined structure by yarns possessed high stretchability (up to 150%). As shown in Figure 4(a), an elbow wrap was sewn with this graphene-based strain sensor. When bending angles were 45°, 90°, and 135°, the sensor produced signals with different intensities (Figure 4(b)). With the increase of bending angles, the relative resistance increased proportionally because of the greater elongation of the sensor. In another work, Afroj et al. reported a dip-coating yarn dyeing technique to produce large-scale graphene-based conductive yarns for textile sensor [71]. Figure 4(c) shows a hank of scoured−bleached 100% cotton white yarn coated with rGO ink and cured at 150°C for 3 min to produce conductive flexible yarns. The knitted sensor by dyed yarns shows an almost 90% repeatable response for bending in forward and reverse directions (Figure 4(d)). This work highlighted the advantage of solution-processed 2D materials and their assembly engineering in fabricating large-scalable strain sensors, because the CVD-grown samples have to be transferred first, and cannot be easily fabricated into highly stretchable, large-scale and complex structure. There are also many other efforts focusing on the structure design, for example, patterning. Lee et al. demonstrated a patterned graphene strain sensor which can monitor small-scale motions [72]. Layer-by-layer assembly was employed to modulate the electrical properties of the sensor. By the increment of graphene thickness, the patterned sensor had enhanced sensitivity. It also had the ability to distinguish subtle motions, such as similar phonations and beats, because the sensor sensitivity can be improved by increasing the pattern density.

Not only graphene and its derivatives but also other 2D materials were explored for pressure or strain sensors. ZnO as a typical piezotronics material together with insulating 2D materials MgO had been employed for this application by Liao et al. [73]. In this work, researchers proposed a structure where ZnO nanorod arrays were at middle part of the piezotronics pressure sensor, while two MgO nanolayers were at the upper and lower levels for electro-tunneling modulation. The ZnO-MgO pressure sensor had an ‘On/Off’ current ratio of 10^5^, sensitivity of 7.1 × 10^4^ gf^−1^ and response time of 128 ms. Based on pressure and strain sensors, touch sensor had also been developed [47]. Hou et al. reported a flexible rGO touch sensor by vacuum filtration and demonstrated that this all-graphene-based flexible touch sensor is capable of discerning the human body from other objects (e.g., plastic, metal, glass, rubber) under ambient conditions. The reason is that the body temperature is much higher than that of surroundings. Therefore, body temperature is an important factor in flexible, wearable sensor design process, and the combination of pressure and temperature in one sensor should be considered in sensor design.

Besides pressure sensors and strain sensors discussed above, gas sensors and biosensors are two kinds of sensors in which 2D materials can also play important roles. The basic principle for 2D materials based gas and biosensors is that due to their large specific surface area, 2D materials can adsorb gas or biomolecules on their surfaces, which will further induce charge transfer and signal change like the sheet resistance [74–77]. For instance, WS_2_ nanoflakes-based ammonia sensors were fabricated through dip-coating method by Li et al. [76]. It demonstrated an increased resistance after exposed to ammonia from 1 ppm to 10 ppm. The response and recovery time of the sensor at 5 ppm ammonia were 120 s and 150 s, respectively. Besides, the sensor has a better adsorption of ammonia compared with some organic interference gases such as ethanol, methanol, acetone, benzene, and formaldehyde. The reason is that ammonia can be physically adsorbed on the monolayer WS_2_ with a moderate degree of charge transfer. Therefore, WS_2_ may be a promising candidate as ammonia sensor [77]. Solution-processed 2D materials are also used in biosensor applications [74]. Blood-based or sweat-based glucose sensors are two typical bio-sensors. By monitoring glucose levels in sweat or blood, researchers can obtain information about the body health. Besides, because the glucose concentration in sweat (0.25–1.5 mM) is much lower than that in blood [78], it is much more difficult to accurately measure the sweat glucose level. To solve this problem, Xuan et al. developed a wearable and flexible sweat glucose sensor based on spray-coating-supported rGO film [79]. This biosensor exhibited the amperometric response to glucose level from 0 mM to 2.4 mM (covers the glucose range in sweat), with a sensitivity of 48 μA/mMcm^2^, a 20s response time, and relatively high linearity (0.99). We also summarize different sensor types with its different 2D materials system in Table 4, which may guide the future senor design by solution-processed 2D materials.

**Table 4.** A summary of different sensors by solution-processed 2D materials.

Material systems	Sensor fabrication	Sensor types		Ref.
Graphene-based	Layer-by-layer	Mechanical sensor	Flexible strain sensor	70,71,72,47
	Dip-coating		Textile sensor	
	Layer-by-layer		Patterned strain sensor	
	Vacuum filtration		Touch sensor	
ZnO-MgO	Spin-coating		Pressure sensor	73
WS_2_ nanoflakes	Dip-coating	Gas sensor	Ammonia gas sensor	76
Graphene-based	Spray-coating	Biosensor	Wearable and flexible sweat sensor	79

### 3.4 Photodetectors and optoelectronics

The principle for photodetectors is absorbing and transferring light to electrical signals including photocurrent or photovoltage. Therefore, the optical properties of materials, especially absorptivity and photoelectrical conversion, are the major factors for photodetectors and optoelectronics. Large number of 2D materials ensure their application in photodetectors because they can absorb light across broad wavelength range from infrared wavelength to deep UV. Optical properties can be widely tuned even in one 2D material, by changing its thickness or adjusting strain, making it suitable for multi-functional optoelectronic devices [35,80]. To be specific, there are many 2D materials having strong interaction with light based on photovoltaic effect, photo-thermoelectric effect, bolometric effect, or photogating effect [81–83]. For the photovoltaic effect, photovoltaic current is generated in the electric fields of junctions between p-type and n-type regions where the induced electron-hole pairs are separated [82]. Photo-thermoelectric effect refers to the hot-carrier-assisted transport phenomenon in which the photo energy can produce hot electrons thus further resulting in a photovoltage. The bolometric effect is also associated with the transport conductance change induced by heating which is produced by the incident photons. Finally, for the photogating effect, which is commonly observed in low-dimensional systems, is considered as that conductance modulation can be achieved by photoinduced gate voltage rather than by trap states [84].

**Figure 5.**
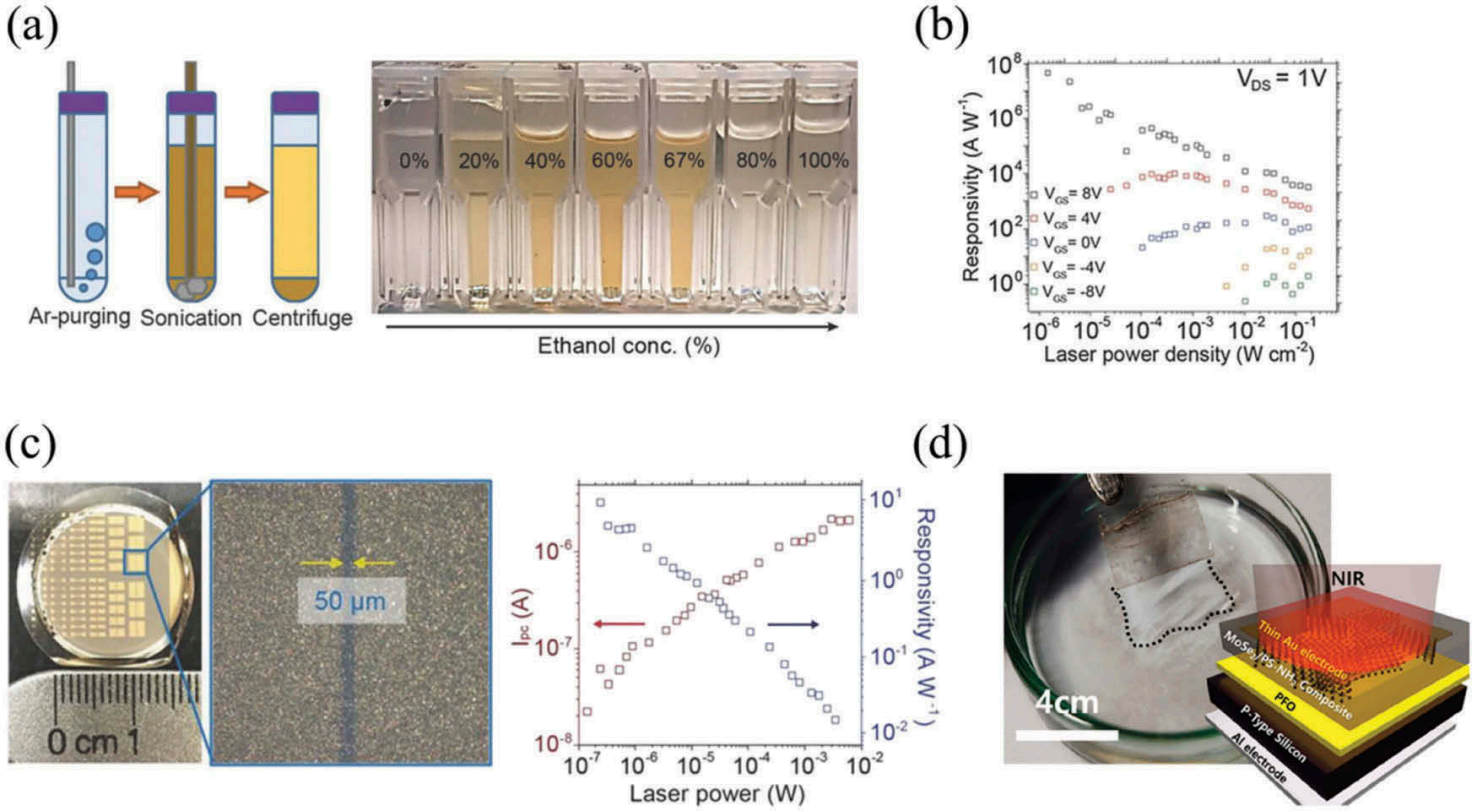
Fabrication and performance of photodetectors from solution-processed 2D materials. (a) Schematic of the preparation process for surfactant-free 2D InSe dispersions and photograph of InSe dispersions for various ethanol concentrations in water. (b) Photoresponsivity plots as a function of laser power density with gate voltage (V_GS_) variations at V_DS_ = 1 V for individual InSe photodetector. (c) Optical image of large-area InSe thin-film devices and power dependence of *I*pc (red) and photoresponsivity (blue) plots. (d) Schematic and process of the p − n diode MoSe_2_ photodetector. (a-c) Reprinted from ref 88. Copyright © 2018 WILEY-VCH Verlag GmbH & Co. KGaA, Weinheim. (d) Reprinted from ref 91. Copyright © 2018 American Chemical Society.

Many photodetectors using 2D materials have been reported and studied. Among them, photodetector by solution-processed 2D materials is a small but promising area. Though liquid-exfoliated 2D materials have not yet been widely employed to fabricate optoelectronic devices because of their relatively poor quality, there are still more and more studies showing their great potential for photoelectronic devices because of their low cost and easy fabrication features. In addition, large efforts have already been made to improve the quality of liquid-exfoliated 2D materials. Through inkjet printing, MoS_2_ dispersion is directly written to form the uniform patterns with high quality (5–7 nm thick) [85]. When the device made by this MoS_2_ dispersion is illustrated by different lights (infrared, white and UV lights), it shows a very rapid photo response which the device current (at V_g_ = 0 V and V_d_ = 1 V) swiftly jumps upwards/downwards and then returns toward the dark current following each On/Off switch. Cunningham et al. reported a thin-film photodetector formed by solution-exfoliated MoS_2_ nano-platelets. The photoresponsivity was about 0.1 mAW^−1^ [86]. Frisenda et al. developed a dielectrophoretic assembly method to assemble TiS_3_ nanosheets and fabricated TiS_3_-based photodetector, with a photoresponsivity of 3.8 mA W^−1^ [87]. Layered indium selenide (InSe) as another kind of 2D material presents many unique properties towards high-performance electronic and optoelectronic device applications. In 2018, Kang et al. employed individual InSe nanosheets in photodetectors [88]. The InSe nanosheets were exfoliated by a surfactant-free, low boiling point, deoxygenated co-solvent system, and they were stabilized by a mixture of ethanol and water (Figure 5(a)). Thus, the product exhibited minimal processing residues and was chemically and structurally pristine, indicating its high-quality compared with the product from traditional surfactant-assisted liquid exfoliation methods. The photodetector fabricated by individual InSe nanosheet exhibited a maximum photoresponsivity of about 5 × 10^7^ A W^−1^, which was almost the highest value among any solution-processed photodetectors to date as shown in Figure 5(b). In addition to individual nanosheet devices, the InSe thin-film photodetectors (Figure 5(c)) fabricated by vacuum filtration demonstrated sublinear dependence of photocurrent (*I*_pc_) and a maximum photoresponsivity of about 10 AW^−1^ (Figure 5(c)), which was higher than the previously reported thin-film photodetectors from solution-processed 2D materials and some from CVD growth 2D materials [86,87,89]. Beyond the works where solution-processed 2D materials were directly used to build photodetectors, 2D materials can also act as decorators. A sensitive and fast monolayer WS_2_ photodetector decorated by liquid-exfoliated SnS nanosheets was fabricated by Jia et al. [90]. The results showed that photoresponsivity of WS_2_ photodetector had been significantly enhanced to 2 A W^−1^ and the response range had been enlarged, after being decorated by liquid-phase exfoliated SnS nanosheets.

The same as other electrical applications, flexible and transparent photodetectors have become a significant consideration. Therefore, solution-processed 2D materials will have more space in this field because they can be easily shaped and patterned onto different substrates. Recently, Hwang et al. used 2D nanosheets to assemble thin-film-based flexible photodetectors as shown in Figure 5(d) [91]. They developed a controlled solvent evaporation method and used it on TMDCs. Based on this method, single or few-layered MoSe_2_ nanosheets modified with the amine-terminated poly(styrene) (PS-NH_2_) can be obtained on the water surface. They finally transferred these layers onto PET substrate to fabricate the flexible p-n junction photodetector. It showed switching time, photoresponsivity, and detectivity were 100.0 ms, 2.5 AW^−1^, and 2.34 × 10^14^ Jones, respectively. Besides, the performance of this PET-based flexible photodetector was comparable with that of the hard Si substrate-based device after 1000 bending cycles, indicating good flexibility of this device. These different devices described above are summarized in Table 5. Due to the wide materials choices and optical property ranges, we believe that in the future, solution-processed 2D materials will make much more contributions to the flexible photodetector.

**Table 5.** A summary of different photodetectors by solution-processed 2D materials.

Material systems	Devices fabrication	Devices characteristics	Ref.
MoS_2_ nanosheets by solvent exfoliation	Inkjet printing	Rise time = 60 ms; Fall time = 570 ms	85, 86
	Based on the LB method	*R =* 0.1 mAW^−1^	
Colloidal TiS_3_ by LPE	Dielectrophoresis assembly	*R =* 3.8 mAW^−1^	87
WS_2_ monolayer by CVD/SnS nanosheets by LPE	SnS sensitizer was drop-cast on the WS_2_ channels.	*R =* 2 AW^−1^ Light/dark signal-to-noise ratio = 10^6^ (under 457 nm excitation)	90
MoSe_2_ nanosheets by PS-NH_2_ assistant sonication exfoliation	Controlled solvent evaporation of MoSe_2_ suspensions spread on water surface	*R =* 2.5 AW−1 Switching time = 100.0 ms Detectivity = 2.34 × 10^14^	91
InSe flakes by LPE	Vacuum filtration	Time response = 3 s *R =* 10 A W^−1^	88

Photoresponsivity: *R*

### 3.5 Flexible electronics

Plenty of applications such as epidermal electronics and wearable electronics need large-area flexible displays. Currently, Si has been largely used in the field of semiconductor devices. Though researchers have found that Si wafers can become flexible when it is thinned down below 25 μm [92,93], the brittle nature largely limits it further application especially in some devices which requires long lifetime and good reliability. Also, there is another type of important flexible electronics materials, the flexible organic semiconductors. However, the relatively poor performance and stability of organic flexible devices increases the resistance of the devices, thus causing higher energy consumption.

Large-area assembled 2D materials would be ideal candidates for future flexible electronic and optoelectronic applications. Previous studies have demonstrated that 2D materials possess excellent mechanical properties. In 2008, Lee et al. measured the elastic properties and intrinsic breaking strength of free-standing monolayer GO membranes. The corresponding Young’s modulus of graphene monolayer is 1.0 ± 0.1 TPa [5]. Subsequently, in 2012, Castellanos-Gomez et al. have measured the elastic properties of freely suspended MoS_2_ nanosheets with 5 to 25 layers which have the average Young modulus of 0.33 ± 0.07 TPa, comparable to that of GO [94]. Stankovich et al. have investigated electrical properties of graphene-based composite materials [95]. They have found that the exfoliated GO nanosheets solution can mix well with polystyrene. After reduction, this graphene-based composite demonstrated a conductivity of 0.1 S m^−1^ at only 1 volume per cent, which was well sufficient for many electrical applications. Li et al. have also reported the mechanical properties of graphene-based composite [96]. They prepared a nacre-like, bio-inspired reduced poly(vinyl alcohol)/GO (R-PVA/GO) composite film via solution-casting. Composite films with 20 wt% PVA loading had tensile strength of 118 MPa and Young’s modulus of 11.4 GPa, which were higher than the pure GO assembled film because of the stronger GO interlayer adhesion from PVA. In addition, reduction is the common approach to make GO conductive. It is found that the conductivity of rGO is not as high as that of intrinsic graphene, which is because the rGO remains oxidized. To elucidate this mechanism, Mattevi et al. investigated the effect of the sp^2^ bonding fraction and residual oxygen in rGO films [97]. It showed that for fully rGO, the oxygen content was 8% (C:O ratio12.5:1) and the sp^2^ concentration was 80%. The sp^2^ fraction of 0.85% would significantly enhance the rGO electrical performance. However, the presence of residual oxygen (8 at. %) greatly hampered the carrier transport and further influenced the conductivity of rGO. Thus, except for the other factor such as flake size or edge configuration, the reduction degree of rGO is also important. All above research guided the flexible device applications of solution-processed graphene and other 2D materials, since 2D materials can be composited in stretchable and conductive polymers to become flexible and conductive composite materials. Also, unlike carbon nanotubes (CNTs), the fabrication process of 2D materials does not require any sorting thus greatly reducing the fabrication complexity. Another big advantage of 2D materials for flexible devices is that there are many 2D materials with different electric properties such as conducting, semiconducting and insulating, which provides wide selection for device optimization.

One of the earliest works using graphene as a flexible transistor was reported by Chen et al. in 2007 [98]. The graphene was mechanically exfoliated from kish graphite onto Si substrates, and then was transferred onto PET substrates based on a lithography-free transfer printing method. This graphene-based flexible transistor has a mobility of 10,000 cm^2^ V^−1^ s^−1^ under ambient conditions. Since that report more works on flexible devices of 2D materials have sprung up. 2D materials prepare by CVD or mechanical exfoliation were mostly used because of their high quality. In 2012, CVD-grown graphene on Ni was transferred onto PDMS first, and then transferred onto a flexible and water-soluble substrate, silk films. The fabricated structure was designed as a bacteria detection for tooth enamel [99]. Such flexible electronic applications also involve press, chemical, and different biological sensors. However, the CVD grown material is small in quantities and mechanical exfoliation method is not applicable to fabricate device arrays despite of its convenience and the less defected products. Solution-processed 2D materials is of great potential in this area because they are high yield and they can be easily assembled into large scale on almost all kinds of substrates.

**Figure 6.**
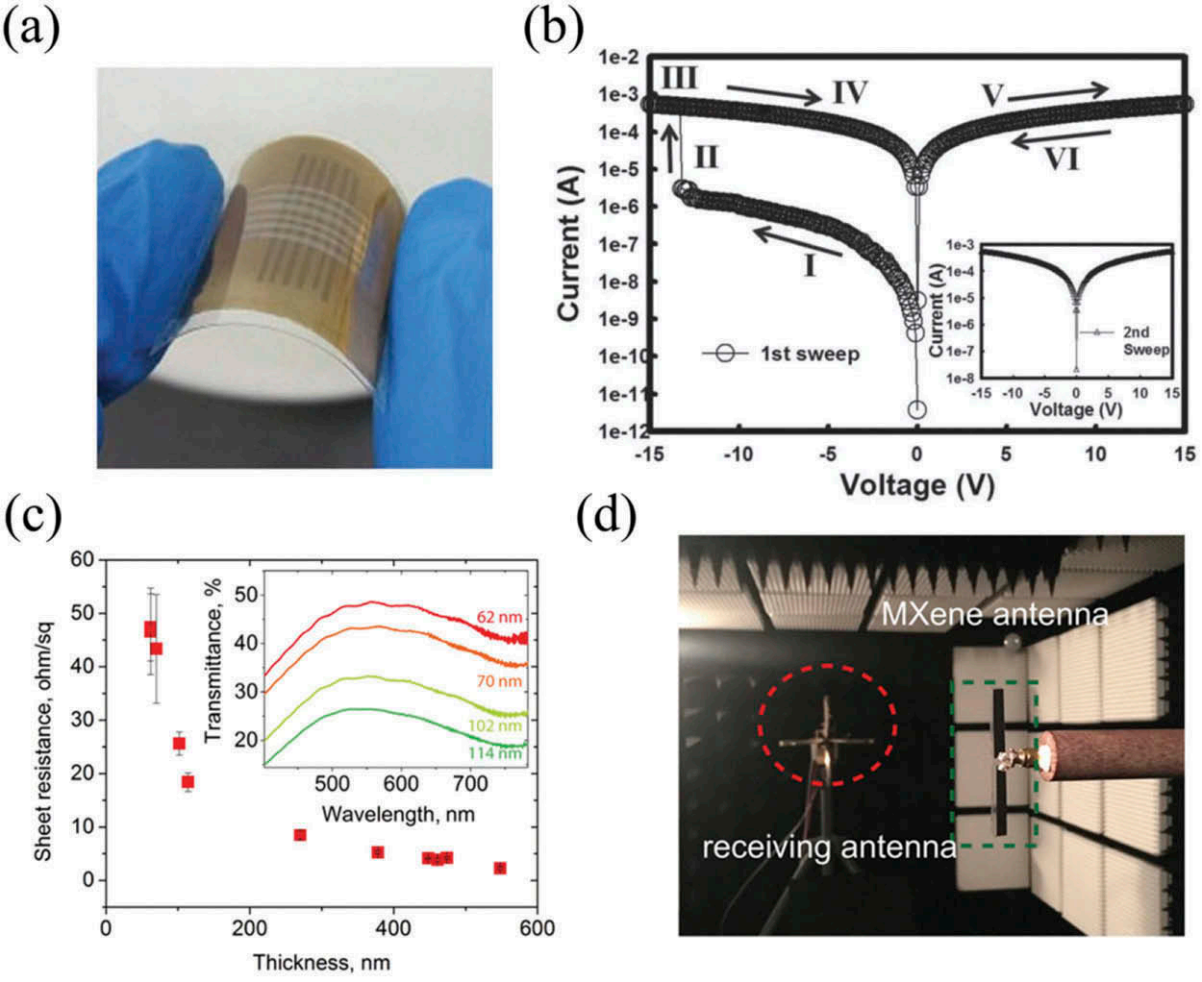
Flexible devices by solution-processed 2D materials. (a) Flexible memory device with sandwich structure of hrGO/lrGO/hrGO. (b) The I–V characteristics of the WORM device. (c) Sheet resistance versus thickness of MXene films. Inset: transmittance in the visible range of antennas with different thicknesses. (d) Photos of an experiment in anechoic chamber to measure the radiation pattern of dipole antennas. (a-b) Reprinted from ref 100. Copyright © 2013 WILEY-VCH Verlag GmbH & Co. KGaA, Weinheim. (c-d) Reprinted from ref 103. Copyright © 2018 American Association for the Advancement of Science.

In this context, an all-carbon flexible memory device by solution-processed graphene has been made [100]. It was a sandwich structure of highly reduced GO (hrGO)/lightly reduced GO (lrGO)/hrGO (Figure 6(a)), which demonstrated electrical bistability with a write-once-read-many-times (WORM) memory effect (Figure 6(b)). In addition, solution-processed 2D TiS_2_ nanosheets were assembled by vacuum-filtration over 25 nm-pore size mixed cellulose ester membranes as the flexible n-type thermoelectric films. During the vacuum-filtration process, chemically exfoliated TiS_2_ nanosheets [101] were homogenously mixed with AlCl_3_ solutions. Multivalent cationic metal Al^3+^ would finally cross-link the nanosheets with each other and forming the TiS_2_ bridges [102]. The resulting flexible TiS_2_ thin films showed a power factor of ∼216.7 *μ*W m^−1^ K^−2^ at room temperature [102]. In 2018, Sarycheva et al. used a one-step spray-coating process to fabricate a translucent and flexible Ti_3_C_2_ MXene antenna for wireless communication [103]. As shown in Figure 6(c,d), the sheet resistance decreased with the thickness. They also showed that the antennas can work well at thicknesses lower than the skin depth of best-known metals or other predicted materials such as copper (1.33 µm of skin depth at 2.4 GHz), silver (1.29 µm of skin depth at 2.4 GHz) and aluminum (1.67 µm of skin depth at 2.4 GHz). Though the development of 2D materials-based flexible devices is still in its early stage, and there are many obstacles need to be solved, the above pioneering demonstrations have already opened door towards this field.

### 3.6. Printed electronics

Printed electronics are electrical devices which are fabricated by various printing techniques. Printed electronics can be fabricated in ambient condition without the need for cleanroom, thus they are cost-effective. It is also capable with flexible electronics and can be fabricated into complicated patterns. The interconnected network structure building in the printed electronics by nano-materials is an important goal in nanoscience. Over the past few years, there are many efforts focusing on the development and selection of new materials for printed electronics, such as organic/inorganic nanoparticles, nanotubes, and nanowires [104,105]. Based on their electrical and mechanical properties, 2D materials are thought to be great candidates toward printed electronic applications. Also, the variety of 2D materials in their electrical and optical properties endows them obvious advantages over organic or inorganic nanostructures [83]. It can be predicted that by selecting different 2D materials as the building blocks on the devices, one can achieve multi-functional design. Further, because ink is of great significance during printing process, 2D materials from solution-processed methods are required. This can not only ensure the stability during printing process but also achieve large-area and low-cost printed electronics.

**Figure 7.**
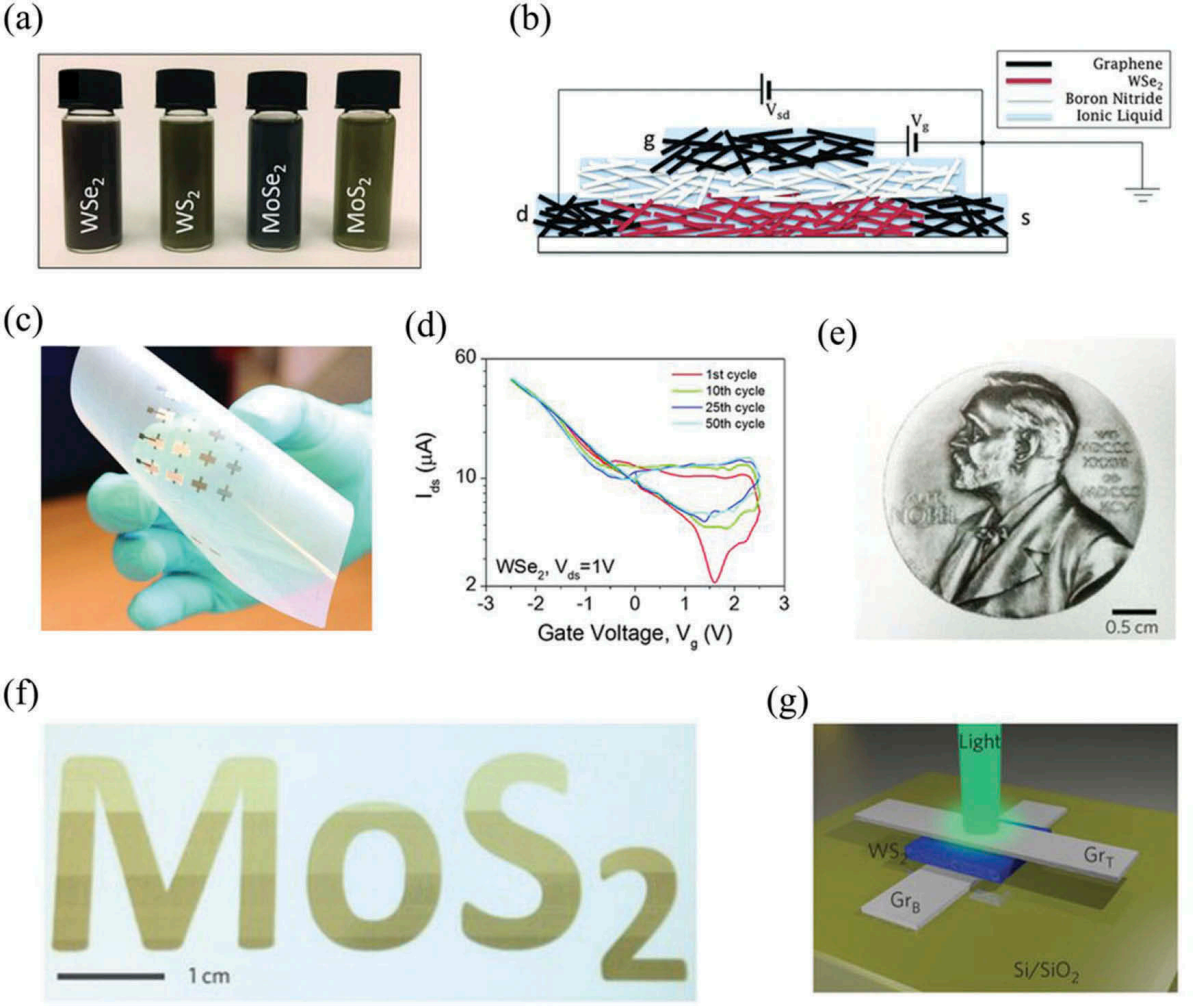
All-printed 2D materials electronics and their properties. (a) Photo of dispersions of MoS_2_, MoSe_2_, WS_2_ and WSe_2_ (C ~ 0.2 mg/ml). (b) Schematic showing all-printed 2D dispersions-based TFT structure. (c) A flexible array of printed TFTs. (d) Transfer curves for a printed TFT with a WSe_2_ active channel after cycling the gate voltage 1, 10, 25, and 50 times. (e) Nobel medal printed with water-based graphene ink on PEL P60 paper. (f) Printed ‘MoS_2ʹ_ with water-based MoS_2_ ink on PEL P60 paper with an increasing number of printed passes (from 1 to 4, top to bottom). Note the relatively good contrast with the paper obtained even with one printed pass, due to the use of a highly concentrated ink. (g) Schematic of an all-printed Gr_B_/WS_2_/Gr_T_ heterostructure on a Si/SiO_2_ substrate. (a-d) Reprinted from ref 10. Copyright © 2017, American Association for the Advancement of Science. (e-g) Reprinted from ref 45. Copyright © 2017, Springer Nature.

Using 2D materials from solution-processed methods as the print inks for large-area printed electronics has become an important branch of applications of 2D materials. Even though it is still at its early stage, several promising works have already been demonstrated [106]. For example, Torrisi et al. have reported a graphene-based ink and used it to print TCFs [106]. The transistor from this ink showed a mobility of about 95 cm^2^V^−1^s^−1^, transmittance of ~80% and sheet resistance of 30 kΩ sq^−1^. This work paved the way toward all-printed 2D material devices. In order to concurrently achieve all the requirements including high electrical conductivity, rapid printing and broad substrate compatibility of graphene inks, Secor et al. employed intense pulsed light annealing to couple inkjet and achieved rapid fabrication of high conductivity (≈25 000 S m^−1^) graphene patterns after a single printing pass [107]. Subsequently, many other types of 2D materials were explored as inks for printed electronics. Kelly et al. fabricated vertically stacked transistors using graphene as the source, drain, gate electrodes, TMDCs (MoS_2_, MoSe_2_, WS_2_, and WSe_2_) as the channel, and h-BN as the separator (Figure 7(a,b)) [10]. The ink suspensions of TMDCs from liquid-phase exfoliation were prepared by sonicating in N-methyl 2-pyrrolidone. After vertical printing process, nanosheets network channels would spontaneously form, which could be fabricated into flexible arrays and displayed an On/Off ratio of 600 and a mobility of about 0.1cm^2^V^−1^s^−1^ (Figure 7(c,d)). It is worth mentioning that the key factor of printed electronics, printable formulations or inks were still far from ideal. Most of them were either low concentration and toxic [106,108,109], or expensive and time-consuming [43,85,110–112]. For the printable ink engineering, there is an important parameter, the inverse Ohnesorge number Z, described as $Z=\sqrt{\gamma \rho \alpha }/\eta$, where η is viscosity, γ is surface tension, ρ is density and α is the set nozzle diameter. Z is used to predict whether a certain kind of ink can form stable drops or not. It has been confirmed that the stable drops can form at the range of 1 < Z < 14. For a nozzle diameter of 21.5 µm, Z of water is approximately 40. Reducing surface tension and increasing viscosity are the two important principles for formulation design. Thus, the exploration and optimization of 2D materials ink have attracted much attention. Some works have already been done to explore suitable inks.

For example, McManus et al. demonstrated a general method and achieved water-based, biocompatible, inkjet-printable 2D materials formulations (Figure 7(e,f)) [45]. In this work, they used two modifier agents to modulate the surface tension and the viscosity of suspension. A non-ionic surfactant, triton X-100, was chosen to reduce the surface tension, and another co-solvent, propylene glycol, was chosen to increase the viscosity as well as improve the biocompatibility of ink. In this general method, they produced water-based, biocompatible and stable WS_2_, MoS_2_, h-BN, and graphene printable inks with a Z value between 20 and 22. Despite Z > 14, the drop in this work was stable. Further, all printable inks can be fabricated as flexible heterostructures and logic memory devices as shown in Figure 7(g). No significant adverse response was found in their study of ink biocompatibility, opening applications of 2D material inks in food, drinks, pharmaceuticals or consumer goods identification tags.

However, inks from solution-processed 2D materials usually suffer from the problem that they are difficult to achieve controlled and stable pattern. The reported Z, which is larger than 14, still limits its further applications, especially when it was used in long printing sessions. To address this challenge, ink formulations using some polymeric binders were reported [110,113]. This approach includes two steps. One is extracting the exfoliated 2D materials from their original dispersions. Another is re-dispersing them in designed solvents with selected polymer binders. Besides lowering Z, this approach can also yield a highly concentrated ink formulation [113]. It is clear that the development of scale-up inks which enables efficient, high-quality and large-scale uniform printing is still a challenge in printed 2D materials electronics. Therefore, high-performance devices can potentially be produced by combining 2D materials with other functional materials such as conducting/semiconducting polymers, biocompatible materials, carbon nanotubes, quantum dots, ferromagnetic or paramagnetic materials.

## 4. Outlook and perspectives

**Figure 8.**
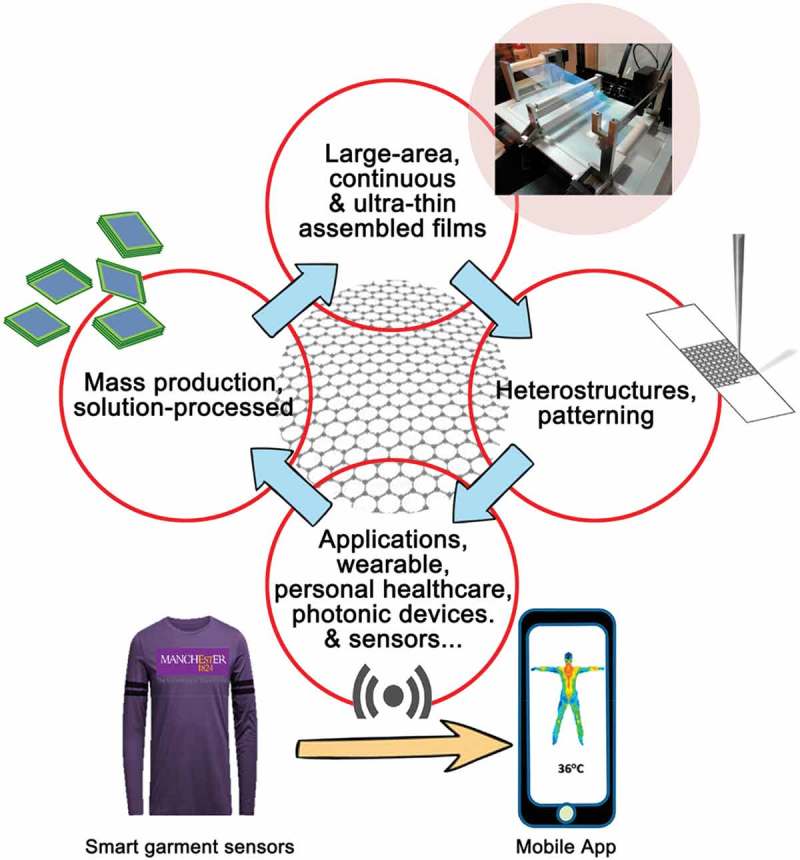
Future research directions to further push the science and applications of solution-processed 2D materials. Reprinted from ref 58. Copyright © 2019 WILEY-VCH Verlag GmbH & Co. KGaA, Weinheim. Reprinted from ref 71. Copyright © 2019 American Chemical Society. Reprinted from ref 72. Copyright © 2017, American Chemical Society. Reprinted from ref 114. Copyright © 2016, American Chemical Society.

In this review, we highlight the importance of solution-processed 2D materials and the corresponding electronics-related applications. We first summarize six typical assembly methods for 2D films preparation, including layer-by-layer method, Langmuir–Blodgett technique, spin coating, electrophoretic deposition method, inkjet printing, and vacuum filtration. In this section, the assembly methods as well as their advantages and disadvantages are carefully discussed. Then, solution-processed 2D materials-based TFTs, TCFs, different sensors, photodetectors, flexible and printed electronics, are discussed. Though they hold significant potential for future electronics development, there still need some efforts to address several key issues. One issue is the imperfection of existing assembly methods. There is no single assembly method which can meet all requirements including cost-effectiveness, simplicity, controllability, scalability and high-quality of assembled film. Thus, the existing assembly processes may need to be reassessed and analyzed. In addition, exploring suitable electrical applications for solution-processed 2D materials and improving the devices performance is another important issue. In the following part, we will propose several directions to which researchers should pay particular attention for further advancing the solution-processed 2D materials and their applications (Figure 8).

The large-area and continuous film assembly is the most important goal for solution-processed 2D materials. Among all assembly methods discussed in this review, we think Langmuir–Blodgett assembly with high controllability is of great promise. To address the large-area production issue, a roll-to-roll dip-coating technique has been developed in the Langmuir–Blodgett process for nanoparticle assembly [114], which in principle could be adopted to 2D materials. Alternatively, to totally explore a new and universal system for continuously depositing or dip-coating the film can be another long-term solution. Besides, in-depth understandings of 2D materials’ behavior during the assembly process also need to be studied. For example, how the properties of 2D materials such as surface charges, sizes, size/thickness aspect ratios, functional groups, and the hydrophilic or hydrophobic properties as well as the interactions between 2D layers and substrates influence the assembly process? These are the basic science behind the assembly process that certainly need more investigations.

For applications, solution-processed 2D materials may not compete with bottom up grown ones in terms of device performance. Thus, special functions or features of devices using solution-processed 2D materials should be explored and maximized. For example, various heterostructures and patterned structures can be readily made via solution process, which is not feasible in bottom up growth. Grand breakthrough might be achieved to make incorporation of functional thin-films based on solution-processed 2D materials to achieve different functions within one device. Furthermore, it is envisioned that wearable, personal health-care devices will be one of the promising applications for solution-processed 2D materials, since 2D flakes possess good mechanical properties, for example, they have high Young modulus of 0.33 ± 0.07 TPa for thin MoS_2_ flakes and [94] and 1.0 ± 0.1 TPa for monolayer graphene [5]. In addition, it has shown that MoS_2_ can be slowly hydrolyzed in aqueous solutions without adverse biological effects [115]. Compared with electrical applications of solution-processed 2D materials-based structures, the exploration of their optical properties and applications is far less than sufficient. It is known that photoluminescence intensity of MoS_2_ is thickness-dependent [116], which makes it suitable for devices like light-emitting diodes and photosensors by engineering the sample thickness. Consequently, the uses of solution-processed 2D materials based photonic devices for optical imaging, light-controlled diagnosis, and therapy in biomedicine may expand the applications of these family of materials and help to accelerate the transition to practical applications from fundamental research.
